# Why Don’t You Go to Bed on Time? A Daily Diary Study on the Relationships between Chronotype, Self-Control Resources and the Phenomenon of Bedtime Procrastination

**DOI:** 10.3389/fpsyg.2018.00077

**Published:** 2018-02-02

**Authors:** Jana Kühnel, Christine J. Syrek, Anne Dreher

**Affiliations:** ^1^Work and Organizational Psychology, Ulm University, Ulm, Germany; ^2^Work and Organizational Psychology, Trier University, Trier, Germany

**Keywords:** procrastination, self-regulation, self-control, chronotype, morningness–eveningness, sleep, diary study

## Abstract

**Background:** This daily diary study investigates the phenomenon of bedtime procrastination. Bedtime procrastination is defined as going to bed later than intended, without having external reasons for doing so. We highlight the role chronotype (interindividual differences in biological preferences for sleep-wake-times) plays for bedtime procrastination. Moreover, we challenge the view that bedtime procrastination is the result of a lack of self-regulatory resources by investigating momentary self-regulatory resources as a predictor of day-specific bedtime procrastination.

**Methods:** One-hundred and eight employees working in various industries completed a general electronic questionnaire (to assess chronotype and trait self-control) and two daily electronic questionnaires (to assess momentary self-regulatory resources before going to bed and day-specific bedtime procrastination) over the course of five work days, resulting in 399 pairs of matched day-next-day measurements.

**Results:** Results of multilevel regression analyses showed that later chronotypes (also referred to as evening types or ‘owls’) tended to report more bedtime procrastination on work days. Moreover, for late chronotypes, day-specific bedtime procrastination declined over the course of the work week. This pattern is in line with expectations derived from chronobiology and could not be explained by trait self-control. In addition, on evenings on which employees had less self-regulatory resources available before going to bed—compared to evenings on which they had more self-regulatory resources available before going to bed—employees showed lower bedtime procrastination. This finding contradicts the prevailing idea that bedtime procrastination is the result of a lack of self-regulatory resources.

**Conclusion:** The findings of this study provide important implications for how bedtime procrastination should be positioned in the field of procrastination as self-regulatory failure and for how bedtime procrastination should be dealt with in practice.

## Introduction

Most people do not get enough sleep on work days (National Sleep Foundation, 2013)^1^ despite sleep’s importance for well-being, performance, and health (Durmer and Dinges, 2005; Hastings et al., 2008; Åkerstedt et al., 2009). A phenomenon held responsible for promoting insufficient sleep on work days is bedtime procrastination. Bedtime procrastination is defined as “going to bed later than intended, without having external reasons for doing so” (Kroese et al., 2016, p. 853), that is, “people just fail to [go to bed]”(Kroese et al., 2014, p. 2). Kroese et al. (2016) argued that this behavior reflects an intention-behavior gap typical for procrastination: Intending to do something, but putting it off despite expecting that it will yield negative consequences. In the case of bedtime procrastination, employees intend to go to bed on time, but put it off despite the negative consequence that they get too little sleep because they have to get up early the next morning. Drawing the parallels between the phenomenon of bedtime procrastination and findings from research on academic procrastination and procrastination at work (Ferrari and Emmons, 1995; Steel, 2007; Kühnel et al., 2016), bedtime procrastination has been conceptualized as self-regulatory failure (Kroese et al., 2016). With this daily diary study, we investigate whether going to bed later than intended is really a matter of poor self-regulation. To answer this question, we investigate the phenomenon of bedtime procrastination on the level of daily processes. We like to provide an alternative explanation for why some people “simply go to bed too late” on work days whereas others do not (Kroese et al., 2016, p. 1): Interindividual differences in underlying biological rhythms that regulate sleep-wake rhythms. More specifically, we investigate how chronotype (Horne and Østberg, 1977; Roenneberg et al., 2003) is related to individuals’ general tendency to go to bed later than intended and how chronotype is related to specific patterns of bedtime procrastination across the work week.

Taken together, this study challenges the view that bedtime procrastination should be conceptualized as a self-regulatory failure (i.e., viewing bedtime procrastination as the result of a lack of self-regulatory resources necessary to go to bed on time). Rather, the phenomenon of bedtime procrastination should be seen as an indicator of a mismatch between employees’ endogenous, biological clocks (that is, chronotype) and societal requirements. In the following, we will pursue two strategies to support our argumentation. First, this study reveals the importance of chronotype for bedtime procrastination. Second, looking at the level of daily processes, we investigate whether day-specific bedtime procrastination is the result of poor momentary self-regulatory resources before going to bed. Results of this study have important consequences for whether and how the construct of bedtime procrastination should be positioned in the nomological network of procrastination. Moreover, practical implications emerge for how to deal with bedtime procrastination that contradict practical implications emerging from previous findings conceptualizing bedtime procrastination as a ‘simple’ self-regulatory failure.

### Chronotype and Bedtime Procrastination

Whether individuals experience difficulties with going to bed as intended should depend on their chronotype. An individual’s chronotype—also referred to as morningness-eveningness (Horne and Østberg, 1976)—represents interindividual differences in preferences for the timing of sleep and wake. Endogenous circadian clocks control humans’ daily rhythms in fundamental aspects of physiology and behavior, among others the timing of sleep and wake (Roenneberg et al., 2003; Czeisler and Gooley, 2007; Hastings et al., 2008). These endogenous, circadian clocks are entrained to the 24-h/day–night cycle primarily driven by sunlight (Duffy and Wright, 2005; Roenneberg et al., 2007b). The exact phase of entrainment is specific for each individual, resulting in a continuum of chronotypes ranging from early ‘larks’ to late ‘owls’ (Roenneberg et al., 2003). Individuals on the one end of the continuum—the early ‘larks’—prefer to go to bed earlier in the evenings and get up earlier in the mornings. Individuals on the other end of the continuum—late ‘owls’—prefer to go to bed later in the evenings and get up later in the mornings (Roenneberg et al., 2003, 2007a). On work free days such as at the weekends and during vacations, individuals can sleep in accordance with their biologically preferred sleep window. Interindividual differences in preferences for the timing of sleep and wake result from an interplay of genetic influences and environmental factors (for twin studies, see for example Hur et al., 1998; Vink et al., 2001) and thus, they cannot be easily overridden by acts of self-control.

For the majority of the population, required wake-up times on work days do not coincide with biologically preferred wake-up times (Roenneberg et al., 2012). On work days, late and intermediate chronotypes have to get up earlier than preferred. As a consequence, they need to use alarm clocks to align their wake-up times with societal obligations (e.g., work and school schedules, Wittmann et al., 2006). As a result of required wake-up times, individuals can only obtain sufficient sleep by going to bed earlier. Simply going to bed earlier, however, is difficult because circadian clocks influence when someone can fall asleep (Strogatz et al., 1987; Lavie, 2001). The circadian drive for wakefulness peaks just before biologically preferred bedtime (Lavie, 1986; Czeisler and Gooley, 2007)—in other words, it is especially difficult to fall asleep in the time frame before biologically preferred bedtime. Thus, especially late chronotypes generate the intention to go to bed earlier or ‘on time’ to obtain sufficient sleep on work days, but eventually, they fail to do so because biological processes do not support the realization of their intention. The later an employee’s chronotype, the more difficulties should the employee face in realizing an earlier bedtime, because the later his/her biological sleep window opens. As a consequence, later chronotypes should be more likely to experience bedtime procrastination on work days.

Hypothesis 1: Later chronotypes indicate, on average across the work week, more bedtime procrastination compared to earlier chronotypes.

The relevance of chronotype for the phenomenon of bedtime procrastination should not only become apparent in mean differences in levels of bedtime procrastination across the work week, but also in *specific patterns* of bedtime procrastination across the days of the work week. The difference in biologically preferred and socially induced sleep timing that occurs between free days and work days is termed ‘social jetlag’ (Wittmann et al., 2006; Roenneberg, 2012) or ‘social sleep lag’ (Kühnel et al., 2016). The term ‘social jetlag’ emphasizes that the difference in sleep timing between free days and work days resembles the situation of traveling across several time zones to West on Friday evenings and flying back on Monday mornings. Similar to when experiencing travel-induced jetlag, individuals experiencing ‘social jetlag’ suffer from symptoms of jetlag that are manifestations of a misaligned circadian system, e.g., problems in sleep, digestion, and performance (Roenneberg et al., 2012). Because of the discrepancy arising between circadian and social clocks at the beginning of the work week, employees experience difficulties in going to bed on time at the beginning of the work week. Especially late chronotypes should have difficulties falling asleep earlier than their biologically preferred time to fall asleep. As a consequence, the later an employee’s chronotype, the more daily sleep is cut short on work days. Thus, over the course of the work week, late chronotypes accumulate a sleep debt, increasing homeostatic sleep drive (Roenneberg et al., 2003). According to the two-process model of sleep regulation (Borbély, 1982; Borbély et al., 2016), the homeostatic sleep drive counteracts the circadian drive for arousal. Accordingly, accumulated sleep debt should facilitate the initiation of sleep even before individual’s biologically preferred time to fall asleep. Thus, as the work week progresses, late chronotypes should be increasingly able to realize their intention to initiate sleep before their biologically preferred time to fall asleep. Therefore, late chronotypes should experience more bedtime procrastination compared to earlier chronotypes (Hypothesis 1), but late chronotypes’ experience of bedtime procrastination should decline over the course of the work week. Put differently, late chronotypes should experience bedtime procrastination especially at the beginning of the work week.

Hypothesis 2: Chronotype and day of the work week jointly predict day-specific bedtime procrastination. The positive relationship between chronotype and bedtime procrastination is stronger on days earlier in the work week compared to days later in the work week.

To prove the unique value of taking chronotype into account when explaining bedtime procrastination, the pattern of bedtime procrastination described above should not be reducible to failed self-regulation. Previous cross-sectional research has established a negative relationship between self-regulation and bedtime procrastination. Individuals who indicated lower values on self-regulation variables (for example, trait self-control resources and impulsivity) reported more bedtime procrastination in general (Kroese et al., 2014, 2016). These cross-sectional results on bedtime procrastination and extensive research conceptualizing dysfunctional procrastination as self-regulatory failure (e.g., Ferrari and Emmons, 1995; Ferrari, 2001; Sirois and Pychyl, 2013) has led researchers to conclude that bedtime procrastination is a form of self-regulatory failure as well. We do, however, want to show that bedtime procrastination is more a matter of individual’s biological rhythm than of a general deficit in self-control. Thus, we hypothesize:

Hypothesis 3: Chronotype and day of the work week jointly predict day-specific bedtime procrastination (Hypothesis 2) even when taking trait self-control into account (that is, even when controlling for main and interactive effects of trait self-control).

### On the Level of Processes: Self-Regulatory Resources and Bedtime Procrastination

Our second approach to support our argumentation that bedtime procrastination should not be conceptualized as self-regulatory failure is to investigate whether self-regulatory resources and bedtime procrastination co-vary over time. In other words: On days on which people lack self-regulatory resources in the evening, are people less able to realize their intention to go to bed on time, that is, are they more likely to experience bedtime procrastination?

Previous research on procrastination *at work* showed that employees are less able to turn their intentions into action on days on which they have less self-regulatory resources at their disposal. Procrastination at work varied from day to day and as a function of employees’ level of self-regulatory resources: On days on which employees had more resources available, they showed less procrastination, compared to days on which employees had less resources available (Kühnel et al., 2016, 2017). In other words, procrastination at work was especially experienced when employees did not have sufficient self-regulatory resources available to initiate an intended course of action. Likewise, if bedtime procrastination would be the result of failed self-regulation and depleted self-regulatory resources, bedtime procrastination should be especially experienced on days on which people do not have sufficient self-regulatory resources available to turn their intention to go to bed on time into action. That is, people should show bedtime procrastination especially on days on which their self-regulatory resources in the evening are depleted, because on these days they are less able to resist temptations and to avoid distractions preventing them from going to bed on time. On days on which people have more self-regulatory resources at their disposal in the evening, however, people should be able to turn their intention to go to bed earlier into action and should experience less bedtime procrastination. Taken together, if bedtime procrastination results from poor self-regulatory resources, a negative within-person relationship should be found between momentary self-regulatory resources and day-specific bedtime procrastination. Because we challenge the notion that bedtime procrastination is a matter of poor self-regulation, we do not expect this negative relationship and investigate the relationship between self-regulatory resources and bedtime procrastination by means of the following research question:

Research question: How is individual’s momentary level of self-regulatory resources in the evening related to day-specific bedtime procrastination on this evening?

## Materials and Methods

### Sample and Procedure

Participants of this daily diary study were employees working in various industries. They were recruited by one of the authors as part of her Master’s thesis. Inclusion criteria for participation was non-shift work, no diagnosed sleep disorder, and at least 70% weekly working time. To motivate employees to participate in the study, participants took part in a lottery where they could win vouchers for an online retailer. This study was conducted in accordance with the model code of ethics of the European Federation of Psychologists’ Associations (European Federation of Psychologists’ Associations [EFPA], 2015). Employees who gave their informed consent to participate filled in several electronic questionnaires. Participants first completed a general electronic questionnaire that assessed sociodemographic characteristics, employees’ chronotype, and trait self-control. In the following work week, participants were asked to answer two daily electronic questionnaires, the first one after getting up in the morning and the second one at the end of the day before going to bed. We used two questionnaires each day to separate measurement occasions of the core predictor variable (momentary self-regulatory resources before going to bed) and the criterion variable bedtime procrastination (Beal, 2015). More specifically, self-regulatory resources were assessed in the evening before going to bed, and bedtime procrastination was assessed after getting up in the morning (on the next day), referring to the experience of bedtime procrastination on the preceding evening. For every questionnaire, participants received an individually scheduled reminder e-mail containing a link to the questionnaire. Of the 133 employees who agreed to participate, three did not fill in any questionnaire, and fourteen had to be excluded due to exclusion criteria of the study (e.g., shift worker, student) or due to incomplete data in the general questionnaire (e.g., participants who failed to answer the general questionnaire). Further, eight employees had to be excluded due to incomplete data in the daily questionnaires (employees needed to provide at least two matched evening-morning-of-the-next-day questionnaire pairs). The final sample comprised 108 employees who, in total, provided data on 510 days, which resulted in 399 matched day-next-day measurements. Thus, final completion rate is 81% for the level of participants^2^. Forty-six percent of the sample was female; average age was 41 years (*SD* = 9.6); and 39% had children. Participants indicated to work, on average, 43 h/week (*SD* = 6.4). Forty percent of the sample had a leadership position. Participants had, on average, 8 years of professional experience in their current organization (*SD* = 9.5).

### General Questionnaire: Measures

#### Chronotype

Employees’ chronotype was assessed with the Munich ChronoType Questionnaire (Roenneberg et al., 2003). The Munich ChronoType Questionnaire determines chronotype based on typical sleep behavior. The questionnaire consists of questions about typical sleep timing on work days and on work-free days. From these data, the midpoint between sleep onset and offset is calculated. Chronotype is defined as the midpoint of sleep on free days, corrected for ‘oversleep’ on free days. Higher values represent a later midpoint of sleep and a later chronotype. For example, a person whose sleep onset and sleep offset on free days are at 12 midnight and at 9 a.m., respectively, has a midpoint of sleep at 4:30 a.m. and a chronotype of 4.5. Midpoint of sleep on free days shows high test–retest reliability (*r* = 0.88, Kühnle, 2006). Moreover, it correlates strongly with sleep logs and wrist actimetry (*r* = 0.92, Kühnle, 2006) and with the biochemical marker melatonin (*r* = 0.89 with dim light melatonin onset, Martin and Eastman, 2002).

#### Trait Self-Control

General self-control was assessed with the German version (Bertrams and Dickhäuser, 2009) of the scale of Tangney et al. (2004), consisting of 13 items. Sample items are “I’m good at resisting temptation” and “People would say that I have very strong self-discipline.” Items had to be answered on a 5-point scale ranging from 1 = *not at all like me* to 5 = *very much like me*. Cronbach’s alpha was 0.82.

### Daily Questionnaire in the Evening before Going to Bed: Measures

#### Momentary Self-Regulatory Resources

Momentary self-regulatory resources before going to bed were assessed with the German version (Bertrams et al., 2011) of the Twenge state self-control capacity scale (Christian and Ellis, 2011). We used the five item short-version of Lanaj et al. (2014). Sample items are “Right now, I feel like my willpower is gone” and “My mind feels unfocused right now.” Items had to be answered on a 5-point scale ranging from 1 = *strongly disagree* to 5 = *strongly agree*. All items were recoded so that higher values represent more self-regulatory resources. Cronbach’s alpha ranged between 0.91 and 0.94 over the days.

### Daily Questionnaire after Getting Up in the Morning: Measures

#### Bedtime Procrastination

Day-specific bedtime procrastination was assessed with six items of the bedtime procrastination scale (Kroese et al., 2016). Items had to be slightly adapted to capture *day-specific* bedtime procrastination on the preceding evening. Sample items are “Yesterday, I wanted to go to bed on time but I just did not do it” and “Yesterday, I was still doing other things when it was time to go to bed.” Items had to be answered on a 5-point scale ranging from 1 = *strongly disagree* to 5 = *strongly agree*. Cronbach’s alpha ranged between 0.87 and 0.88 over the days.

#### Control Variable Unfinished Tasks before Going to Bed

Unfinished tasks before going to bed on the preceding evening were assessed with the following two items adapted from Syrek and Antoni (2014): “I couldn’t complete many of my tasks yesterday so now I need to finish them today” and “I am discontent that I didn’t manage to complete yesterday’s important tasks.” Items had to be answered on a 5-point scale ranging from 1 = *strongly disagree* to 5 = *strongly agree*. The correlation between the two items ranged between *r* = 0.55 and *r* = 0.68 over the days.

## Results

### Descriptive Statistics

**Table 1** shows means, standard deviations, intercorrelations between variables, and intraclass correlations (ICCs). For all day-specific variables, we ran null models with the Hierarchical Linear Modelling (HLM) 7.01 software package (Raudenbush et al., 2011) to calculate the proportions of variance that were within-person and between-person. All day-specific variables showed substantial day-to-day variation (within-person variance): 75% of the variance in bedtime procrastination, 51% of the variance in self-regulatory resources before going to bed, and 55% of the variance in unfinished tasks before going to bed resided at the within-person level.

**Table 1:** Means, standard deviations, and correlations of variables.

Variable	*M*	*SD*	1-ICC^d^	1	2	3	4	5	6	7	8
(1) Day-specific bedtime procrastination	2.68	1.09	0.75		0.17**	0.01	0.00				
(2) Momentary self-regulatory resources before going to bed	3.35	0.98	0.51	–0.12		–0.25***	0.01				
(3) Day-specific unfinished tasks in the evening	2.25	0.95	0.55	0.23*	–0.36***		–0.03				
(4) Time (day of the work week)^a^	1.51	1.09	–	–0.06	0.18	–0.10					
(5) Chronotype	3.94	1.00	–	0.18	–0.16	–0.26*	0.05				
(6) Trait self-control	3.41	0.57	–	–0.11	0.27**	–0.41***	0.10	–0.22*			
(7) Age	40.54	9.61	–	–0.19*	0.37***	–0.10	–0.03	–0.37***	0.19		
(8) Gender^b^	0.46	0.50	–	–0.05	–0.21*	0.02	0.07	–0.01	–0.05	–0.27**	
(9) Leadership position^c^	0.40	0.49	–	–0.01	0.38**	–0.07	0.04	–0.11	0.18	0.21*	–0.34***

*^*a*^0 = Monday, 1 = Tuesday, 2 = Wednesday, 3 = Thursday. Correlations below the diagonal are person-level correlations. To calculate these person-level correlations, day-level data below the diagonal were averaged across days (N = 108). Correlations above the diagonal are day-level correlations (N = 399). Above the diagonal, variables 1, 2, and 3 were centered around the respective person-mean. ^*b*^Gender: 1 = female, 0 = male. ^c^Leadership position: 1 = yes, 0 = no.*^*d*^Intraclass correlation (ICC) = ratio of the between-person variance to the total variance, 1-ICC = ratio of the within-person variance to the total variance. *^∗^p < 0.05, ^∗∗^p < 0.01, ^∗∗∗^p < 0.001.*

The within-person correlations among the day-specific variables (shown above the diagonal in **Table 1**) show that bedtime procrastination was positively related to self-regulatory resources before going to bed (*r* = 0.17, *p* < 0.01). The between-person correlations below the diagonal in **Table 1** show that bedtime procrastination was negatively related to age (*r* = -0.19, *p* < 0.05) and positively related to employees’ general level of unfinished tasks (*r* = 0.23, *p* < 0.05).

### Analytic Strategy

We conducted multilevel analyses with the HLM 7.01 software package (Raudenbush et al., 2011). For these analyses, we coded day of the work week into the variable ‘time’ (0 = *Monday*, 1 = *Tuesday*, 2 = *Wednesday*, 3 = *Thursday*). Day-level predictor variables self-regulatory resources and unfinished tasks were centered around the respective person mean (group-mean centering) because we were interested in how an employee’s day-specific state and experiences—in comparison to his or her state and experiences on other days—predict bedtime procrastination. Person-level predictor variables chronotype and trait self-control were centered around their grand-mean.

To predict day-specific bedtime procrastination, we specified and compared several nested hierarchical linear models (see **Table 2**). In Model 1, we entered the day-level predictor variable time (day of the work week) and the person-level predictor variable chronotype (Hypothesis 1). We followed best practice recommendations of Aguinis et al. (2013) and built a random intercept and random slope model (Model 2) as a prerequisite for testing cross-level interactions in the following models. In Model 3, we tested the cross-level interaction of chronotype on time (Hypothesis 2). In Model 4, we entered the day-level predictor variable self-regulatory resources before going to bed (Research question). Both Models 5a and 5b show robustness tests of our findings. In Model 5a, we entered the day-level control variable unfinished tasks in the evening to investigate whether unfinished tasks were related to bedtime procrastination. Furthermore, we entered trait self-control and we tested the cross-level interaction of trait self-control on time (Hypothesis 3). Thus, we investigated relationships between chronotype, time and bedtime procrastination, controlling for trait self-control. In Model 5b, results of a model are depicted in which the cross-level interaction of chronotype is omitted. This model allows the investigation of the relationships between trait self-control, time and bedtime procrastination without taking chronotype into account.

**Table 2:** Results of multilevel analyses predicting day-specific bedtime procrastination.

	Null model			Model 1			Model 2			Model 3		
	Est	*SE*	*t*	Est	*SE*	*t*	Est	*SE*	*t*	Est	*SE*	*t*
Intercept	2.681	0.071	37.84***	2.688	0.096	27.96***	2.689	0.100	26.74***	2.695	0.100	26.94***
Time (day of the work week)^a^				–0.004	0.043	–0.10	–0.005	0.044	–0.11	–0.008	0.043	–0.18
Self-regulatory resources (at bedtime)												
Unfinished tasks in the evening												
*Level 2 predictors*												
Chronotype				0.118	0.070	1.67 ^†^	0.105	0.070	1.49	0.297	0.101	2.91**
Trait self-control												
*Cross-level interactions*												
Time^a^ × Chronotype										–0.118	0.045	–2.63**
Time^a^ × Trait self-control												
–2 × log likelihood	1170.918			1168.135			1167.52135			1160.697		
Δ -2 × log likelihood (*df*)					2.783	(2)		0.613	(2)		6.823	(1)^∗∗^
Level 1 Intercept Variance (*SE*)		0.891	(0.073)		0.890	(0.073)		0.872	(0.088)		0.862	(0.087)
Level 2 Intercept Variance (*SE*)		0.296	(0.076)		0.285	(0.074)		0.387	(0.159)		0.384	(0.157)
Level 2 Slope Variance (*SE*) – Time^a^								0.011	(0.035)		0.006	(0.032)
Level 2 Intercept-Slope Covariance (*SE*)								–0.041	(0.062)		–0.036	(0.060)
		**Model 4**			**Model 5a**			**Model 5b**		
		**Est**	***SE***	***t***	**Est**	***SE***	***t***	**Est**	***SE***	***t***
		
Intercept		2.696	0.098	27.31***	2.694	0.097	27.67***	2.689	0.097	27.54***
Time (day of the work week)^a^		–0.008	0.043	–0.19	–0.008	0.042	–0.18	–0.006	0.043	–0.14
Self-regulatory resources (at bedtime)		0.214	0.077	2.77**	0.244	0.079	3.06**	0.259	0.079	3.25**
Unfinished tasks in the evening					0.061	0.079	0.77	0.063	0.079	0.79
*Level 2 predictors*										
Chronotype		0.284	0.100	2.82**	0.250	0.101	2.47*	0.103	0.072	1.42
Trait self-control					–0.291	0.174	–1.67^†^	–0.337	0.173	–1.94^†^
*Cross-level interactions*										
Time^a^ × Chronotype		–0.109	0.044	–2.46*	–0.095	0.045	–2.10*			
Time^a^ × Trait self-control					0.123	0.078	1.57	0.156	0.077	2.00*
–2 × log likelihood		1153.116			1149.260			1153.633		
Δ -2 × log likelihood (*df*)			7.581	(1)^∗∗^		3.855	(3)		13.888	(4)^∗∗^
Level 1 Intercept Variance (*SE*)			0.840	(0.084)		0.832	(0.084)		0.834	(0.084)
Level 2 Intercept Variance (*SE*)			0.374	(0.153)		0.352	(0.149)		0.357	(0.150)
Level 2 Slope Variance (*SE*) – Time^a^			0.005	(0.031)		0.004	(0.031)		0.010	(0.031)
Level 2 Intercept-Slope Covariance (*SE*)			–0.030	(0.058)		–0.022	(0.057)		–0.027	(0.058)

*Est = estimate. ^*a*^0 = Monday, 1 = Tuesday, 2 = Wednesday, 3 = Thursday. Model 5b is compared to nested Model 2. ^†^p < 0.10, ^∗^p < 0.05, ^∗∗^p < 0.01, ^∗∗∗^p < 0.001.*

### Test of Hypotheses

Model 1 shows that chronotype tended to be positively related to bedtime procrastination, but that the estimate failed to reach significance (Estimate = 0.118, *SE* = 0.070, *t* = 1.67, *p* = 0.09). Later chronotypes tended to indicate, on average across work days, more bedtime procrastination compared to earlier chronotypes. Hypothesis 1 was not supported.

**FIGURE 1:**
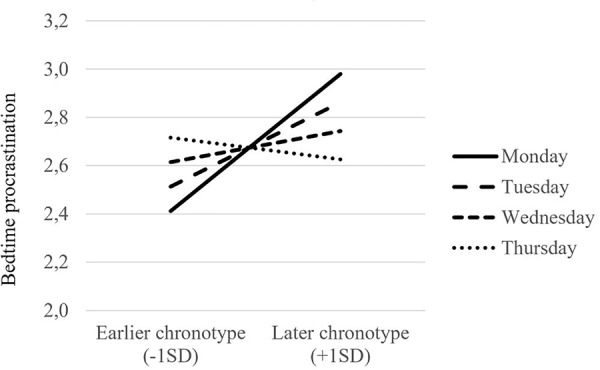
Cross-level interaction of chronotype with time (day of the work week) on day-specific bedtime procrastination.

To test Hypothesis 2 that the positive relationship between chronotype and bedtime procrastination is stronger on days earlier in the work week compared to days later in the work week, Model 3 tested whether chronotype and time (day of the work week) jointly predicted day-specific bedtime procrastination. Model 3 showed that chronotype was a significant cross-level moderator of the relationship between day of the work week and bedtime procrastination (Estimate = -0.118, *SE* = 0.045, *t* = -2.63, *p* < 0.01). The pattern of the interaction is depicted in **Figure 1**. It shows that the positive relationship between chronotype and bedtime procrastination was stronger on days earlier in the week. We tested the significance of the simple slopes with the computational tool by Preacher et al. (2006). The simple slope of chronotype predicting bedtime procrastination was significant on Mondays (simple slope = 0.28, *SE* = 0.10, *t* = 2.82, *p* < 0.01) and Tuesdays (simple slope = 0.17, *SE* = 0.08, *t* = 2.30, *p* < 0.05), and not significant on Wednesdays (simple slope = 0.06, *SE* = 0.07, *t* = 0.88, *p* = 0.38) and Thursdays (simple slope = -0.05, *SE* = 0.09, *t* = -0.49, *p* = 0.63). Taken together, Hypothesis 2 was supported.

The aim of Model 4 was to answer the research question whether self-regulatory resources before going to bed are related to bedtime procrastination. Model 4 showed that self-regulatory resources before going to bed were a significant *positive* predictor of bedtime procrastination (Estimate = 0.214, *SE* = 0.077, *t* = 2.77, *p* < 0.01). Thus, on evenings on which employees indicated to have *more* self-regulatory resources at their disposal—compared to evenings with less self-regulatory resources at their disposal—employees showed *more* bedtime procrastination. Thus, results contradict the idea that day-specific bedtime procrastination is the result of depleted self-regulatory resources.

Models 5a and 5b address the question whether trait self-control accounts for our findings (Hypothesis 3). Model 5a showed that trait self-control could not account for the significant relationship between chronotype, time (day of the work week) and bedtime procrastination. After entering trait self-control as a cross-level moderator on the relationships between time and bedtime procrastination, this interaction was not significant (Estimate = 0.123, *SE* = 0.078, *t* = 1.57, *p* = 0.12) and chronotype was still a significant moderator of the relationship between time and bedtime procrastination (Estimate = -0.095, *SE* = 0.045, *t* = -2.10, *p* < 0.05). When trait self-control was entered as main effect only, chronotype was still a significant moderator of the relationship between time and bedtime procrastination as well (not depicted in **Table 2**, Estimate = -0.109, *SE* = 0.044, *t* = -2.45, *p* < 0.05). Thus, trait self-control could not account for the significant relationship between chronotype, time and bedtime procrastination, supporting Hypothesis 3.

Model 5b showed that quite the opposite seems to be true, that is, that chronotype seems to account for the relationship between trait self-control and bedtime procrastination. When considering the cross-level interaction of trait self-control on time *without* taking chronotype into account, this interaction was significant (Estimate = 0.156, *SE* = 0.077, *t* = 2.00, *p* < 0.05). The interaction patterns showed that—especially on days earlier in the work week—trait self-control was negatively related to bedtime procrastination. Once chronotype was taken into account (Model 5a), this interaction was not significant any more. Thus, chronotype seems to—at least partly—account for the relationship between trait self-control, time (day of the work week) and bedtime procrastination.^3^

### Robustness Tests

Because bedtime procrastination is defined as “going to bed later without having external reasons for doing so,” we tested whether having unfinished tasks in the evening is related to bedtime procrastination (Model 5a). Unfinished tasks might be “external reasons” to delay bedtime. Model 5a showed that unfinished tasks in the evening were neither significantly related to bedtime procrastination (Estimate = 0.061, *SE* = 0.079, *t* = 0.77, *p* = 0.44), nor did the inclusion of unfinished tasks change the results.

Further, we like to rule out the alternative explanation that bedtime procrastination on the evening of the *preceding* day is a third variable explaining the positive relationship between self-regulatory resources before going to bed and bedtime procrastination on the current day. One might speculate that bedtime procrastination results in shorter sleep and subsequently, in both less self-regulatory resources and in greater sleep pressure on the evening of the next day, so that employees are less likely to procrastinate their bedtime. We repeated our analyses and took into account bedtime procrastination of the preceding day as a control variable (not depicted in **Table 2**). Results remained unchanged. Thus, we can rule out that bedtime procrastination on the preceding day is a third variable creating a spurious relationship between self-regulatory resources before going to bed and bedtime procrastination.

## Discussion

This daily diary study showed that bedtime procrastination—defined as going to bed later than intended, without having external reasons for doing so—is related to interindividual differences in biological preferences for sleep-wake-times, that is, chronotype. Later chronotypes (also referred to as evening types or ‘owls’) tended to report more bedtime procrastination than earlier chronotypes (also referred to as morning types or ‘larks’). Day-specific bedtime procrastination showed a specific pattern over the course of the work week that was in line with expectations derived from chronobiology. More specifically, for late chronotypes, day-specific bedtime procrastination declined over the course of the work week. Moreover, results of this study do not support the idea that bedtime procrastination is solely the result of a lack of self-control: Neither did trait self-control incrementally contribute to the explanation of the specific pattern of bedtime procrastination over the course of the work week, nor did employees experience bedtime procrastination especially on days on which their self-regulatory resources were depleted before going to bed. On the contrary, on evenings on which employees indicated to have *more* self-regulatory resources at their disposal—compared to evenings with less self-regulatory resources at their disposal—employees showed *more* bedtime procrastination. These findings do not support the idea that people don’t go to bed on time because they lack self-regulatory resources necessary to put their intention into action.

Taken together, results of this study suggest an alternative perspective on previous between-person findings on the phenomenon of bedtime procrastination that led researchers to conclude that bedtime procrastination is a matter of failed self-regulation. In line with previous research that found negative relationships between bedtime procrastination and indicators of self-control resources (*r* = -0.52 and *r* = -0.39 in Kroese et al., 2014, and Kroese et al., 2016, respectively), we found negative (but small) between-person relationships between trait self-control and bedtime procrastination (*r* = -0.11) and between self-regulatory resources before going to bed and bedtime procrastination (*r*_between-person_ = -0.12) as well. That is, people who indicated to have less self-control in general experienced more bedtime procrastination than people who indicated to have more self-control in general. We suggest that low self-control could be seen as an emerging, rather than as an explaining, phenomenon. That is, instead of making low self-control responsible for why people delay their bedtime, having lower self-control resources available may emerge from the combination of societal, environmental demands (among them, required wake-up times on work days) and individuals’ biological preferences (chronotype). Later chronotypes are more likely to be forced to live in misalignment with their biological preferences (Wittmann et al., 2006). Living in circadian misalignment might continuously put demands on people’s self-regulation, what might exhaust their self-control resources in the long run (becoming apparent in unhealthy lifestyle habits and obesity; see Wittmann et al., 2006; Urbán et al., 2011; Roenneberg et al., 2012).

Continuous demands on late chronotypes’ self-regulation and consequently, exhausted self-control resources, may explain why late chronotypes show more general behavioral procrastination, that is, procrastination across a range of tasks and life domains. In a cross-sectional study, Digdon and Howell (2008) showed that students who had an evening preference (late chronotypes) reported more general procrastination compared to students who had an intermediate or morning preference (early chronotypes). Similarly, Ferrari et al. (1997) showed that ‘trait procrastinators’ were more likely to claim that they are more alert and active during the late afternoon and evening hours (late chronotype) and less likely to claim that they are more alert and active during the morning hours (early chronotype). In a study on general behavioral procrastination by Sirois et al. (2015), chronotype was not assessed, but results showed that general behavioral procrastination was significantly related to shorter sleep duration, longer time needed to fall asleep and more extensive use of medication to fall asleep, all of which can be interpreted as indicators for circadian misalignment which more likely affects late chronotypes. Thus, it would be an interesting avenue for future research to explore whether the experience of general behavioral procrastination can be partly explained by individual’s chronotype and potential deficits in self-control resources arising from circadian misalignment (for relationships between circadian misalignment and procrastination at work, see Kühnel et al., 2016, 2017).

What consequences do our findings have regarding how bedtime procrastination should be positioned to general behavioral procrastination, that is, procrastination across a range of tasks and life domains? We agree with Kroese et al. (2016) that bedtime procrastination is an experience that can be conceptualized as an intention-behavior gap, because people form the intention to go to bed on time and—especially late chronotypes—fail to do so. However, results of our study suggest that bedtime procrastination is different from procrastination of tasks in the academic or work context not only because these intention-behavior gaps appear in different contexts, but because the underlying mechanisms may be different. We suggest that people do not fail to realize their intention (Tonight, I go to bed earlier!) because they are unable to control their desire for short-term, pleasurable experiences or benefits (Ferrari and Emmons, 1995; Tice and Baumeister, 1997; Sirois and Pychyl, 2013), but because biological processes do not support the realization of their intention. Steel (2007) defined dysfunctional behavioral procrastination as the *voluntary* delay of an intended course of action despite expecting to be worse off because of the delay. The endogenous, circadian drive causes later chronotypes to feel more alert in the evening than earlier chronotypes. This may prompt later chronotypes to engage in activities other than going to bed although they know they may be worse off because their daily sleep is cut short. Thus, one might question whether bedtime procrastination—as it is currently conceptualized—is indeed a *voluntary* delay. We like to pick up a suggestion made by one of the reviewers that it might be fruitful to limit bedtime procrastination to instances in which the person could go to bed and fall asleep but does voluntarily delay going to bed.

Results for our research question showed that people do not fail to go to bed on time because they lack self-regulatory resources necessary to put their intention into action. On the contrary, on evenings on which employees indicated to have *more* self-regulatory resources at their disposal, employees delayed their bedtime. Why did we find this positive within-person relationship between self-regulatory resources and bedtime procrastination? One might speculate that on evenings on which people have more resources at their disposal, they are less dependent on restoring their resources via sleep. That is, on these evenings, people may have the feeling that they can afford to go to bed later. Similarly, on evenings on which people feel able to exert self-control, they may decide to use their resources to address other issues instead of going to bed on time. The next morning, however, they may regret this decision and indicate that they did not go to bed on time. Besides these speculations, an alternative explanation for the positive within-person relationship between self-regulatory resources and bedtime procrastination is that the measure of momentary self-regulatory resources partly captures tiredness (Bertrams et al., 2011). On evenings on which employees indicate to have less self-regulatory resources at their disposal, they feel more tired and thus, they show less bedtime procrastination, because they are able to realize their intention to go to bed on time. However, research has shown that having less self-regulatory resources is distinct from being tired (Vohs et al., 2011). Moreover, our additional analyses in which we controlled for bedtime procrastination on the preceding evening suggest that tiredness—that potentially arises from bedtime procrastination on the preceding evening—does not seem to explain our findings. Nevertheless, future research might further explore the positive within-person relationship between self-regulatory resources in the evening and bedtime procrastination, for example by assessing tiredness in the evening and by taking it into account.

A limitation of our study is that we took into account work days only. It may be an interesting avenue for future research to investigate the phenomenon of bedtime procrastination on evenings preceding work-free days, as well. We propose that on evenings preceding work-free days, late chronotypes may not form the intention to go to bed early or ‘on time’ because they do not necessarily have to get up early the next day. Consequently, late chronotypes should experience less bedtime procrastination on evenings preceding work-free days compared to evenings preceding work days. Thus, future research on bedtime procrastination might want to include work-free days as well.

Another interesting avenue for future research would be to investigate work-related consequences of bedtime procrastination. Do outcomes relevant for organizations and employees such as, e.g., day-specific affect and job performance co-vary with bedtime procrastination over the course of the work week? In other words: Does day-specific bedtime procrastination result in impairments in, e.g., affect and job performance on the next day? In addition, future research may explore differential patterns in fluctuations in affect (Larsen and Kasimatis, 1990) and job performance over the course of the work week as a function of employee’s chronotype.

### Practical Implications

We do not want to depict individuals who experience difficulties with going to bed ‘on time’ as victims of their circadian rhythms. To a certain extent, circadian entrainment processes can be supported to avoid further delays of the biologically preferred sleep window. Entrainment processes rely on environmental cues (‘zeitgebers’) of which light is the most important one (for a review, see Duffy and Wright, 2005). Greatly simplified, circadian entrainment theory suggests that lack of light during the day (for example, due to working indoors) and exposure to light in the early night may result in phase delays (that is, shifts in timing to a later hour) for most people (Duffy and Wright, 2005; Wright et al., 2013). Especially exposure to light with short wavelength (blue light) seems to be effective at suppressing melatonin (Lockley et al., 2003). Thus, we recommend that individuals who experience difficulties with going to bed ‘on time’ should avoid exposure to blue light in the early night (emitted by electronic devices such as, e.g., e-readers, tablets, and mobile phones). Several apps are available that reduce the screen’s emission of blue light during preset times such as the evening and the night. However, using the mobile phone or tablet in bed is not unreservedly advisable even when a blue light filter is enabled. Using electronic devices may be an activating activity that prevents employees from mentally detaching themselves from work issues what may impede subsequent sleep (Sonnentag et al., 2008). Especially late chronotypes are at risk to enter a vicious cycle, because they may be tempted to use electronic devices in bed because they may feel less ready to sleep when going to bed on work days (Fossum et al., 2014). Unfortunately, being exposed to blue light and to potentially activating content may further delay sleep-onset. Thus, especially late chronotypes should be educated about this issue and they should reconsider using electronic devices in bed. Moreover, we like to point out recommendations by researchers of the Division of Sleep Medicine at Harvard Medical School (2007). To foster restful sleep, they recommend to form healthy sleep habits such as limiting bed room activities to sleep and sex only. Exercising 3 h before bedtime, heavy food intake before bedtime, and caffeine (found in coffee, tea, chocolate, cola and some medicines) intake 4 to 6 h before bedtime should be avoided. Because struggling to fall asleep may lead to frustration—what can make it even more difficult to fall asleep—they recommend to get out of bed in these instances, to go to another room, and to do something relaxing until one is feeling tired enough to sleep. Taken together, our answer to the question we started with (‘Why don’t you go to bed on time?’) is that individual’s chronotype plays a prominent role for bedtime procrastination, but that measures can be taken to avoid further delays of individual’s biologically preferred bedtime.

## Ethics Statement

In Germany, approval by an ethics committee is only necessary if medical or physiological information about the participants are collected or when participants are treated in a way that may harm their health. As we did not use such kind of data or apply such kind of treatment in our study, we did not need to get approval from the Ethics Committee. However, we closely followed the guidelines for the treatment of human subjects of the German Psychological Association (DGPs, 2016), and this study was conducted in accordance with the model code of ethics of the European Federation of Psychologists’ Associations (EFPA; 2015). That is, we informed participants about purpose, duration, and procedures of the study and about the confidentiality of all the data that were collected. Furthermore, participants were informed that they could always withdraw from the study without any disadvantages, about incentives of participation, and whom they could contact about questions. After participants had received this information they agreed to participate in our study and also that data from them was collected, stored, and analyzed for our research. Deutsche Gesellschaft für Psychologie e. V. (DGPs), Berufsverband Deutscher Psychologinnen und Psychologen e.V. (2016). Berufsethische Richtlinien. Available: https://www.dgps.de/fileadmin/documents/Empfehlungen/berufsethische_richtlinien_dgps.pdf/ [Accessed 2016]. European Federation of Psychologists’ Associations (EFPA) Board of Ethics (2015). Model code of ethics [Online]. Available: http://ethics.efpa.eu/guidelines/ [Accessed 2016].

## Author Contributions

JK and AD made substantial contributions to the conception and design of the study. AD collected the data. JK analyzed and interpreted the data and drafted the manuscript. All authors (JK, CS, and AD) revised the manuscript for important intellectual content.

## Conflict of Interest Statement

The authors declare that the research was conducted in the absence of any commercial or financial relationships that could be construed as a potential conflict of interest.

## Footnotes

1 The National Sleep Foundation’s International Bedroom Poll includes data from the United States, Canada, Mexico, the United Kingdom, Germany, and Japan. In these countries, between 58% (Canada and United Kingdom) and 70% (Japan) of respondents indicated that they get less sleep than needed on work days.

2 The participants who had to be excluded due to missing data did not differ from the final sample, neither with regard to demographic variables [gender: *t*(114) = -0.48; *p* = 0.633; age: *t*(113) = -0.94. *p* = 0.347; leadership position: *t*(114) = 2.04, *p* = 0.071] nor with regard to our variables of interest [chronotype: *t*(114) = -0.25, *p* = 0.805, bedtime procrastination: *t*(114) = -0.71, *p* = 0.500, trait self-control: *t*(114) = -1.37, *p* = 0.174].

3 We find the same pattern of results when testing Models 5a and 5b excluding the Level-1 predictors self-regulatory resources and unfinished tasks in the evening. In this alternative Model 5a, Time × Chronotype was significantly related to bedtime procrastination (Estimate = -0.108, *SE* = 0.046, *t* = -2.36, *p* < 0.05) while Time × Trait self-control was not significantly related to bedtime procrastination (Estimate = 0.099, *SE* = 0.078, *t* = 1.27, *p* = 0.206). In the alternative Model 5b, Time × Trait self-control tends to be positively related to bedtime procrastination (Estimate = 0.135, *SE* = 0.078, *t* = 1.73, *p* = 0.086). To further corroborate that our data supports Hypothesis 3, we rely on the work of MacKinnon et al. (2000) on the equivalence of the mediation, confounding, and suppression effect. We tested whether chronotype ‘confounds’ the relationship between trait self-control and the slope of time predicting bedtime procrastination. In our case, we hypothesized that chronotype should operate like a ‘confounder,’ because this means that chronotype is responsible for/can explain the relationship between trait self-control and the slope of time predicting bedtime procrastination. We computed a Sobel test to investigate whether the ‘confounding’ effect of chronotype is significant. Results indicate (Sobel’s *z* = 2.065, *SE* = 0.019, *p* < 0.05) that chronotype significantly ‘confounds’ the relationship between trait self-control and the slope of time predicting bedtime procrastination. This means that the relationship between trait self-control and the slope of time predicting bedtime procrastination can be explained by chronotype to a significant extent, supporting Hypothesis 3.
